# *Plasmodium falciparum* histidine-rich protein 2 and 3 genes deletion in global settings (2010–2021): a systematic review and meta-analysis

**DOI:** 10.1186/s12936-022-04051-7

**Published:** 2022-01-29

**Authors:** Ayalew Jejaw Zeleke, Asrat Hailu, Abebe Genetu Bayih, Migbaru Kefale, Ashenafi Tazebew Amare, Yalewayker Tegegne, Mulugeta Aemero

**Affiliations:** 1 Department of Medical Parasitology, School of Biomedical and Laboratory Sciences, College of Medicine and Health Sciences, University of Gondar, Gondar, Ethiopia; 2 School of Medicine Addis, Ababa University, Addis Ababa, Ethiopia; 3 Armauer Hansen Research Institute, Addis Ababa, Ethiopia; 4 Department of Pediatrics, School of Medicine, College of Medicine and Health Sciences, University of Gondar, Gondar, Ethiopia

**Keywords:** Histidine-rich protein, Deletions, Systematic review, Meta-analysis

## Abstract

**Background:**

The usefulness of histidine-rich protein-2/3 (HRP2/3)-based rapid diagnostic tests of malaria due to *Plasmodium falciparum* has been threatened by the appearance of mutant PfHRP2/3 genes. This study was undertaken to determine the global pooled estimates of PfHRP2/3gene deletions.

**Methods:**

Relevant publications were identified from electronic databases such as; PubMed, EMBASE, and MEDLINE online. Besides, all the relevant literatures were retrieved through Google and Google Scholar. STATA software was used for data analysis. The pooled estimates were calculated using random effect model. The summary estimates were presented using forest plots and tables.

**Results:**

A total of 27 studies were included in the systematic review. However, only 24 and 17 studies were included for PfHRP2 and 3 gene deletion meta-analysis, respectively. The prevalence of PfHRP2 gene deletion across the individual studies ranged from the highest 100% to the lowest 0%. However, the meta-analysis result showed that the global pooled prevalence of PfHRP2 and PfHRP3 gene deletions were 21.30% and 34.50%, respectively. The pooled proportion of PfHRP2 gene deletion among false negative PfHRP2-based RDTs results was found to be 41.10%. The gene deletion status was higher in South America and followed by Africa. The pooled estimate of PfHRP2 gene deletion among studies, which did not follow the WHO PfHRP2/3 gene deletion analysis protocol was higher than their counter parts (21.3% vs 10.5%).

**Conclusions:**

This review showed that there is a high pooled prevalence of PfHRP2/3 gene deletions in *Plasmodium falciparum* confirmed isolates and also a high proportion of their deletions among false-negative malaria cases using PfHRP2-based RDT results. Hence, malaria diagnosis based on PfHRP2-based rapid tests seems to be less sensitive and warrants further evaluation of PfHRP2/3 gene deletions.

## Background

Malaria kills several thousands of people globally. Since 2010, the World Health Organization (WHO) guidelines has stated that parasite-based diagnosis of malaria should be confirmed before treatment is given [1]. Although quality assured microscopy remains the gold standard for diagnosis of symptomatic malaria, rapid diagnostic tests (RDT) are playing an important role in malaria case management. The use of RDTs has grown substantially since they were first developed in the 1990s. RDTs are currently used in the public healthcare sector in 90 malaria endemic countries. The cost-effectiveness, minimal training requirement of health centre staff, and availability of results within a few minutes make RDTs the preferred tools in resource limited settings. The wide scale introduction of RDTs has contributed to the substantial decline in malaria burden witnessed in the last two decades globally [2].

Detection of malaria by RDTs is principally based on identification of one or more of three antigens, i.e., histidine-rich protein-2 (HRP2), lactate dehydrogenase (LDH), and aldolase [3, 4]. Of these, HRP2 is used for specific detection of *Plasmodium falciparum* because of its exclusive expression in this species of human *Plasmodium* at asexual and sexual phases in the blood stage infection [5–8], while the others are pan-specific. Increasing reports on variable test performances of PfHRP2-based RDT in different endemic regions and different tests on panels of blood samples targeting PfHRP2 [9] is of concern, and has been attributed to several factors including parasite factors (such as parasite density, quantity of parasite antigen produced or its persistence in peripheral blood and variability of target epitopes in antigen structure) [10–12]. Of these, lack of the PfHRP2 gene in the parasite species resulting in no expression of the corresponding antigen is the most important factor [12, 13]. Parasites that do not express the HRP2 protein can cause false-negative results by PfHRP2-based RDTs [14].

The HRP2 protein has an epitope that shows cross-reactivity with HRP3**.** Therefore, PfHRP2-based RDTs sometimes detect infections in PfHRP2**-**deleted parasites due to the presence of HRP3, especially at higher parasite densities. However, the absence of both HRP2 and HRP3 antigens renders the parasites undetectable by HRP2-based RDTs**.**

The deletions of PfHRP2 and PfHRP3, which are among the potential causes of false negative reports of RDT have been reported in populations from Peru [15], Mali [16], India [17] and in a clinical cases from Brazil [18]**.** Moreover, false-negative RDT results due to PfHRP2/3 gene deletions have been reported in 10 sub-Sahara African countries [19]. It is possible to predict that the continued use of only PfHRP2 RDTs will quickly select for parasites without the PfHRP2 gene [20]. This phenomenon occurs due to selection of infections caused by parasites lacking the PfHRP2 gene, which will subsequently contribute more towards onwards transmission than wild-type parasites that are correctly diagnosed due to the expression of PfHRP2.

Some countries, with high proportion of RDT false-negative results due to HRP2 gene deletions, have decided to change national diagnostic guidelines [21, 22]. However, before undertaking any drastic changes in diagnostic testing policies, malaria programmes need robust epidemiological data about local PfHRP2/3 deletion prevalence. The WHO recommends the use of non PfHRP2-based RDTs if the estimated proportion of *P. falciparum* cases with false-negative HRP2 RDT results due to PfHRP2/3 deletions is above 5%. If the estimated proportion is less than 5% countries are advised to establish a monitoring scheme whereby studies are repeated in two years if the 95% confidence interval does not include 5%, or within one year if it includes 5% [23]. Therefore, comprehensive estimates of the extent of PfHRP2/3 gene deletions are needed for the programmatic management of malaria within the context of global malaria control programs and hence, this systematic review and meta-analysis aimed to assess the current global status of PfHRP2/3 gene deletions.

## Methods

### Study design

Systematic review and meta-analysis was conducted according to preferred reporting items for systematic reviews and meta-analyses guidelines (PRISMA) [24]. The PRISMA checklist was used to ensure inclusion of relevant information in the analysis.

### Search strategy

A comprehensive search was conducted to identify relevant published articles in the last 12 years (2010–2021) about the prevalence of PfHRP2/3 gene deletions from *P. falciparum* isolates in different parts of the world. Electronic data base searches were carried out systematically for including essential studies. PubMed, African Journal of Medline, Google, Google scholar and other potential sources were used to retrieve data. The search terms were used separately and in combination using Boolean operators like “OR” or “AND.” An example of keywords used in PubMed to select relevant studies was as follows: (prevalence) OR (prevalence) [MeSH Terms] AND (*plasmodium falciparum*) OR (*Plasmodium falciparum*) [MeSH Terms] AND (HRP2/3 gene deletion) OR (HRP2/3 gene deletion) [MeSH Terms] AND (*P. falciparum* histidine rich protein 2/3) OR (P*. falciparum* histidine rich protein 2). Moreover, a snowball search was used to search the citation lists of included studies. The EndNote software version X7 was used to manage references and remove duplicated references.

### Exclusion and inclusion criteria

Observational studies (cohort, retrospective and cross-sectional studies) that described the prevalence of PfHRP2/3 gene deletions among *P. falciparum* confirmed isolates were included. All included studies were original research articles published in English and peer-reviewed journals between January 2010 and April 2021. Moreover, articles which had sample size of  ≥ 37, and an acceptable quality (score  ≥ 6) were included in the meta-analysis. *P. falciparum* confirmed isolates which are confirmed using only microscopy were not included.

### Data extraction

Two authors (AJZ, YT) performed data extraction using excel spreadsheet form. A third reviewer (MA) arbitrated any discrepancies between the two authors. From each study, the following parameters have been extracted: first author’s name, year of publication, study country/continent, study population, year of data collection, study design, sample size, diagnostic method of malaria, type of HRP gene investigated (PfHRP2 and/or PfHRP3), the number of variants with deletions in PfHRP2/3 genes among false-negative PfHRP2-based RDT results.

### Study selection and quality assessment

Studies were assessed for quality, with only high-quality studies included in the analysis. The quality of included studies has been assessed in accordance with Newcastle–Ottawa quality assessment scale [25]. Two authors (AJZ, YT) independently assessed the methodological quality, quality of reported data (extractable data to calculate the pooled prevalence of PfHRP2/3 gene deletions), stratified data on the types of PfHRP (2 and 3) and clear data research design of the included studies. After assessing the quality of each included study on the basis of these criteria, a composite quality score was assigned, ranging from 0 to 9. Studies scoring 6 and above were judged to be of high quality.

### Data management and analysis

Data were entered into excel and then exported to the open meta-analyst software (Stata version 11 software package (Stata Corporation, College Station, TX)) for performing meta-analysis and descriptive analyses. A random effect model was used to calculate the pooled estimates of PfHRP2/3 gene deletions across studies. Point estimation with a confidence interval of 95% was used. Sensitivity analysis was conducted to assess the role of each study in the overall prevalence estimation. The presence of publication bias was assessed by using Egger’s test; p  < 0.05 was considered indicative of statistically significant publication bias. Trim and fill method analyses have been conducted to obtain a bias-adjusted effect estimate. Heterogeneity across studies was checked by Cochran’s *Q* statistic and *I*^2^ statistics. Subgroup analysis was performed based on type of PfHRP, gene deletion analysis approaches, and continent since there have unexplained significant heterogeneity.

## Results

### Selection of studies included in the systematic review and meta-analysis

**Fig. 1:**
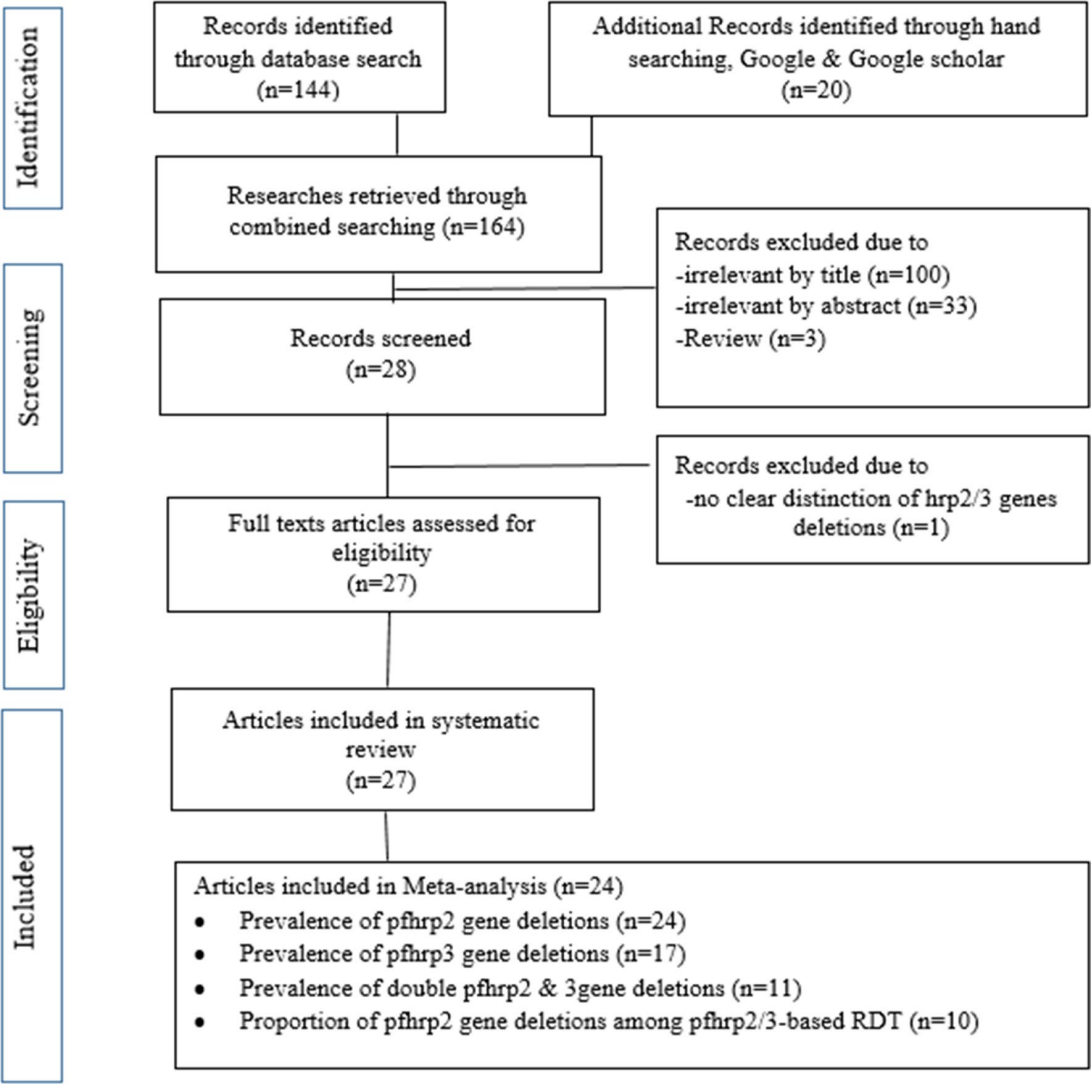
PRISMA chart of the selection steps of included studies

A total of 164 studies were identified during the analysis as presented in the PRISMA flowchart (Fig. 1). Twenty seven of 164 were screened after removing most of the records due to their irrelevancy following critical reviewing of titles and content of their abstracts. However, only 24, 17, and 10 studies were included for meta-analysis in order to calculate the estimated values of PfHRP2, PfHRP3 and double gene deletions, respectively. Moreover, only 10 studies were included in the estimation of the pooled proportion of PfHRP2 gene deletion among PfHRP2-based RDT false negative cases.

### Characteristics of studies included in the systematic review and meta-analysis

A total of 27 studies are included in the systematic review, however, only 24 and 17 studies were included for PfHRP2 and 3 gene deletion analysis. Out of the 27 studies, 18 were from African countries (Mali, Senegal, Congo, Ghana, Eritrea, Rwanda, Kenya, Mozambique, Zambia, Swaziland, Nigeria, Djibouti, Angola, Uganda, Ethiopia, Sudan and South Sudan). The remaining 6 and 3 studies were from South America and Asian countries, respectively. The blood samples in the studies were collected from symptomatic and/or asymptomatic patients between 1996 and 2018 years. A total of 7576 samples with confirmed *P. falciparum* isolates were included for PfHRP2/3 gene deletion analysis (Table 1). Based on the systematic review analysis, the prevalence of PfHRP2 gene deletion ranged from the lowest 0% to the highest 100% [26, 27].

**Table 1:** Characteristics of studies included in the systematic review and meta-analysis

Authors	Country	Continent	Study population	Sample collection Time	Study design	No. study population
Koita et al. [16]	Mali	Africa	Patients	1996	Retrospective	480
Wurtz et al. [38]	Senegal	Africa	Febrile patients	2009–11	Cross sectional	122
Parr et al. [36]	Congo	Africa	Asymptomatic	2013–14	Cross sectional	783
Amoah et al. [39]	Ghana	Africa	Healthy children	2015	Cross sectional	94
Menegon et al. [40]	Eritrea	Africa	Symptomatic	2013–14	Cross sectional	144
Kozycki et al. [41]	Rwanda	Africa	Symptomatic	2014–15	Cross sectional	156
Beshir et al. [19]	Kenya	Africa	Asymptomatic	2014	Cross sectional	106
Gupta et al. [42]	Mozambique	Africa	All population	2010–16	Cross sectional	69
Berhanie et al. [22]	Eritrea	Africa	Symptomatic	2016	Cross sectional	50
Kobayashi et al. [43]	Zambia	Africa	Asymptomatic	2009–17	Retrospective	36
Funwei et al. [44]	Nigeria	Africa	Febrile children	^b^	Not specified	66
Plucinski et al. [45]	Angola	Africa	Febrile patients	2016	Cross sectional	466
Iriart et al. [30]	Djibouti	Africa	Patients	2019	Cross sectional	79
Golassa et al. [27]	Ethiopia	Africa	Febrile patients	2015	Cross sectional	50
Bosco et al. [46]	Uganda	Africa	Febrile patients	2017–19	Cross sectional	195
Prosser et al. [47]	^a^	Africa	Patients	2006–08	Retrospective	210
Alemayehu et al. [48]	Ethiopia	Africa	Febrile patients	2018	Cross sectional	218
Ranadive et al. [26]	Swaziland	Africa	Symptomatic	2012–14	Cross sectional	1353
Pati et al. [49]	India	Asia	Febrile patients	2013–16	Cross sectional	384
Bharti et al. [50]	India	Asia	Febrile patients	2014	Cross sectional	1521
Kumar et al. [17]	India	Asia	Febrile patients	2010	Cross sectional	48
Viana et al. [51]	^a^	S. America	Febrile patients	2010–11	Cross sectional	223
Gamboa et al. [13]	Peru	S. America	Febrile patients	^b^	Retrospective	148
Goes et al. [52]	Brazil	S. America	Febrile patients	2016–17	Retrospective	159
Akinyi et al. [53]	Peru	S. America	Patients	1998–2005	Retrospective	188
Solano et al. [12]	Colombia	S. America	Patients	1999–2009	Retrospective	100
Fontecha et al. [54]	^a^	S. America	Febrile patients	2011–17	Cross sectional	128

^a^Multiple countries

^b^Not specified

### Heterogeneity and publication bias

**Fig. 2:**
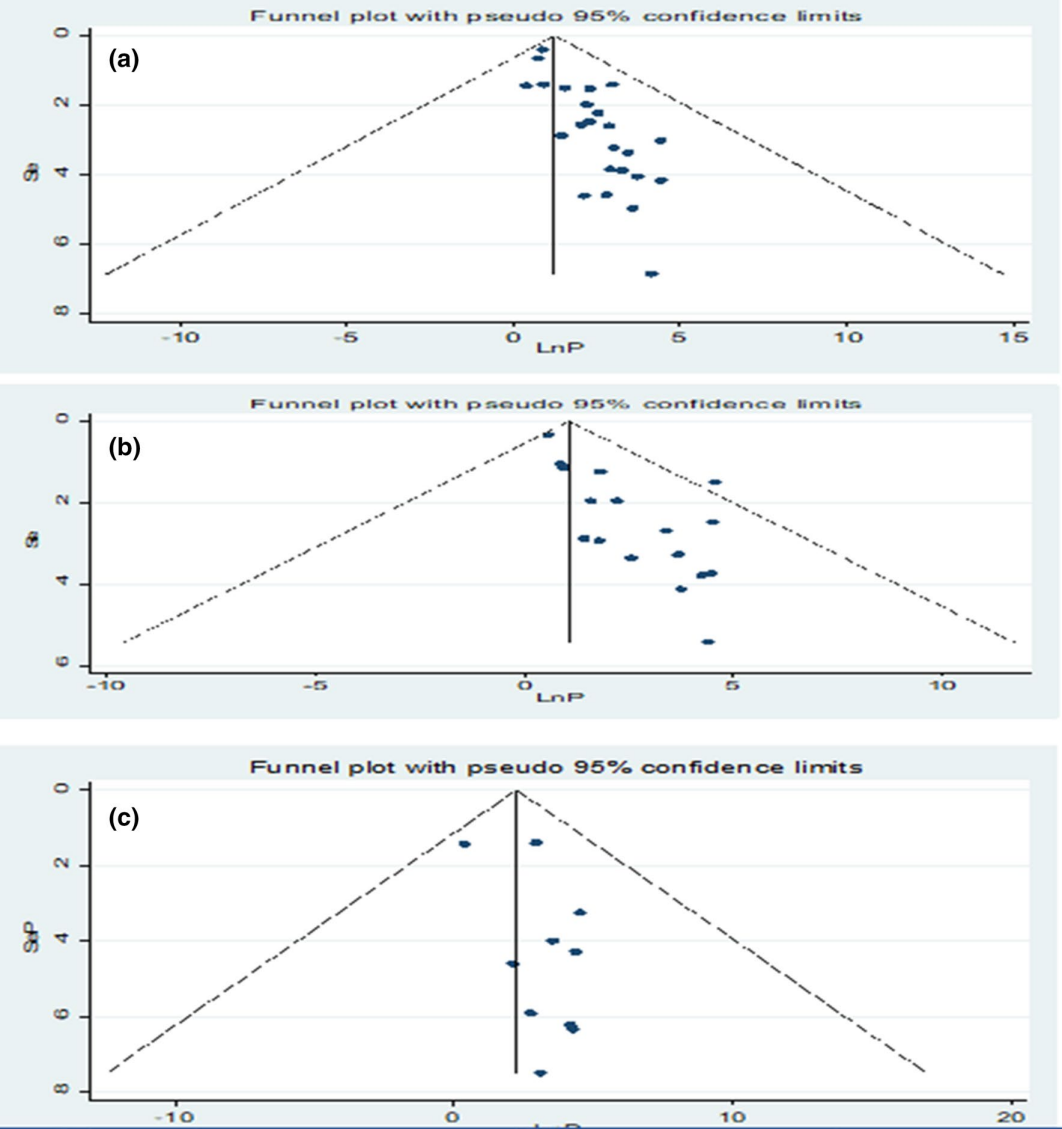
Funnel plot for determination of pooled prevalence of *Pfhrp* 2 (**a**), *Pfhrp3* (**b**) among *P. falciparum* isolates and pooled proportion of *Pfhrp2* gene deletion among false negatives using *Pfhrp2*-based RDTs (**c**)

The existence of heterogeneity and publication bias was determined within the included studies. Consequently, there were considerable heterogeneity across the included studies in this meta-analysis (*I*^2^  > 75%). The included studies were assessed for potential publication bias using Egger’s test. Separate analyses using Egger’s test for the pooled prevalence of PfHRP2/3 gene deletion estimation among confirmed *P. falciparum* isolates and for pooled proportion calculation among false negatives using PfHRP2-based RDTs were done. The Egger’s test for publication bias was not significant (p  < 0.05) for the proportion determination, but there was evidence of publication bias within included studies for the prevalence estimation. Adjusting the findings using the trim and fill method would provide a bias-adjusted effect estimate. Therefore, trim and fill analysis was carried out. However, the bias-adjusted effect for pooled prevalence estimate of PfHRP2 and 3 gene deletions was somehow different compared with the results presented below indicating estimates minimally impacted by publication bias. Moreover, the presence or absence of potential bias was assessed visually by funnel plot. The funnel plots indicated that our meta-analysis is less impacted by publication bias since all (100%) of the studies fell within the triangular region (Fig. 2).

### Sensitivity analysis

**Fig. 3:**
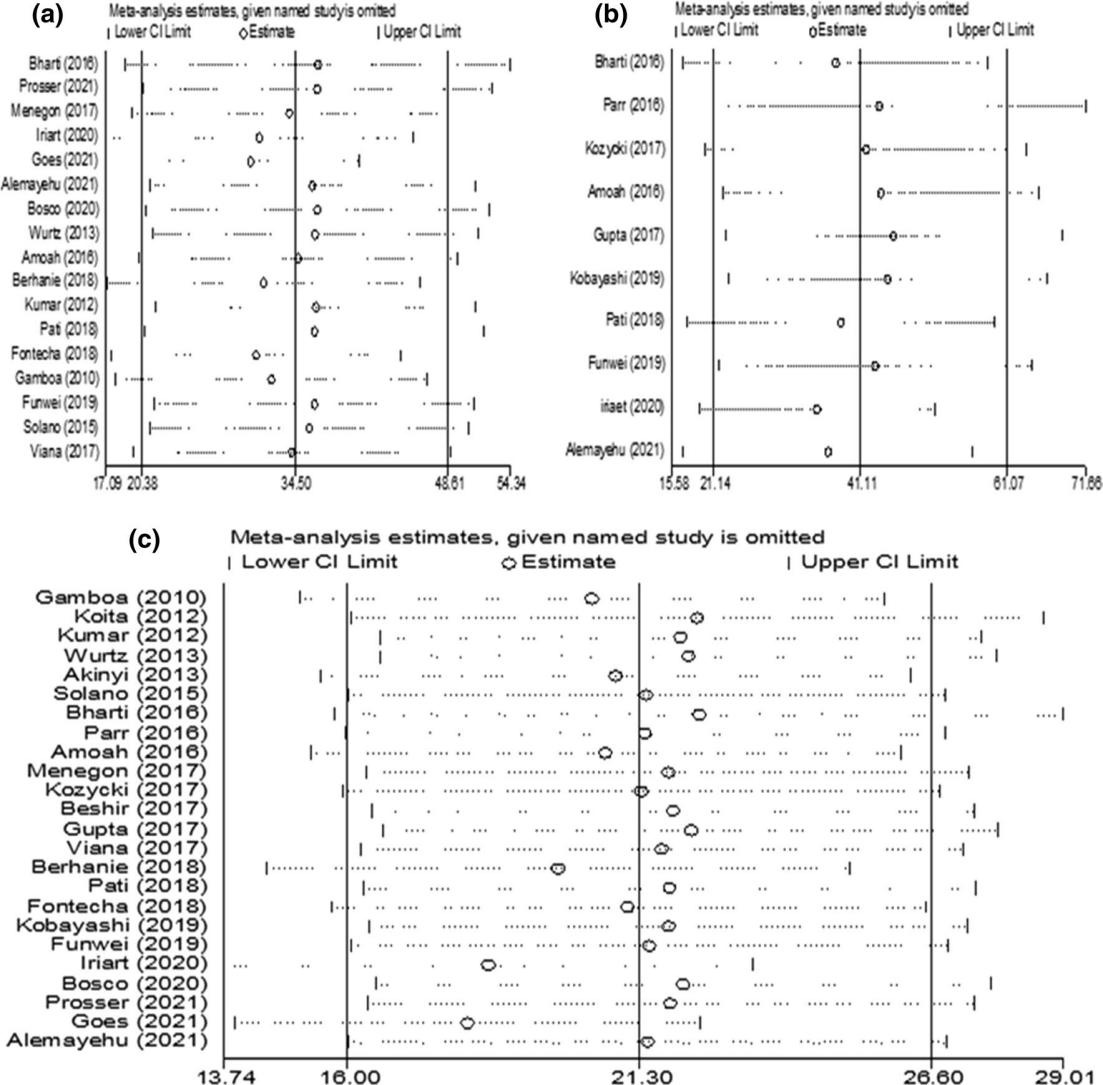
Sensitivity analysis for prevalence of *Pfhrp3* (**a**) and *Pfhrp2* (**c**) deletion estimation; **b** shows for proportion of *Pfhrp2*-calculation

Sensitivity analysis showed that the effect of individual studies on pooled estimate was insignificant, suggesting the robustness of an aggregated estimate. Therefore, the pooled prevalence of PfHRP2/3 among *P. falciparum* isolates and the pooled proportion of PfHRP2 gene deletions among false negatives using PfHRP2-based RDTs were steady and reliable when examined by neglecting one study at a time (Fig. 3).

### Pooled prevalence of PfHRP2/3 gene deletions

**Fig. 4:**
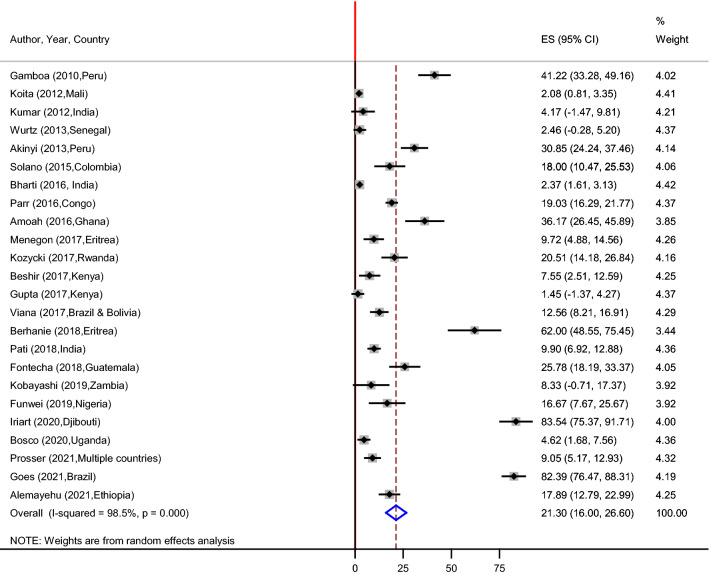
Forest plot representing pooled estimates *Pfhrp2* gene deletion across studies from different parts of the World

**Fig. 5:**
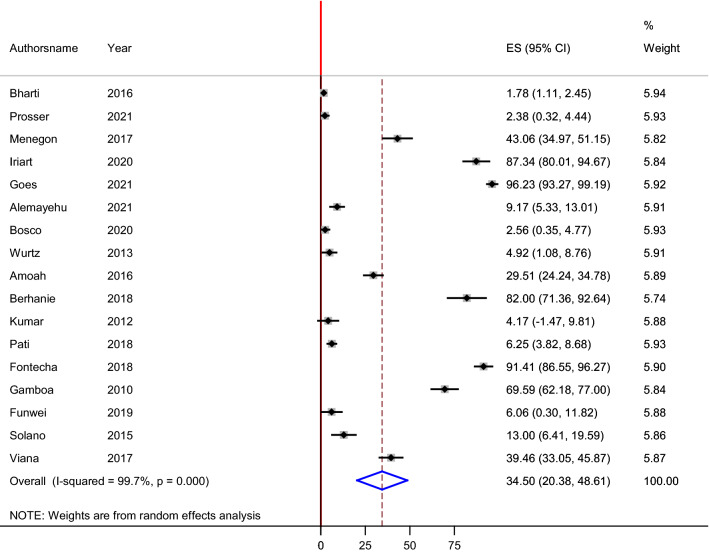
Forest plot representing pooled estimates *pfhrp3* gene deletion across studies from different parts of the World

Out of 27 published articles that are included in this systematic review, twenty four studies were included for the overall global estimation of PfHRP2 gene deletion. Three studies were excluded by the STATA software analysis due to their outlier characteristics. The estimated pooled prevalence of PfHRP2 gene deletion using random effects model was found to be 21.30% (95% CI 16.00–26.60%). The lowest and highest pooled estimated values for the prevalence of PfHRP2 gene deletions were reported in Kenya (1.45%) and Djibouti (83.54%), respectively (Fig. 4). On the other hand, seventeen studies were included for the pooled prevalence of PfHRP3 gene deletion analysis. Nine, five and three of the studies were from Africa, South America and Asia continents, respectively. The highest and lowest estimated values were recorded in Brazil (96.23%) and India (1.78%), respectively. The pooled prevalence of parasites with deletions in PfHRP3 gene was 34.50% (95% CI 20.38–48.61%) (Fig. 5).

### Subgroup analysis of PfHRP2/3gene deletion by continent

**Fig. 6:**
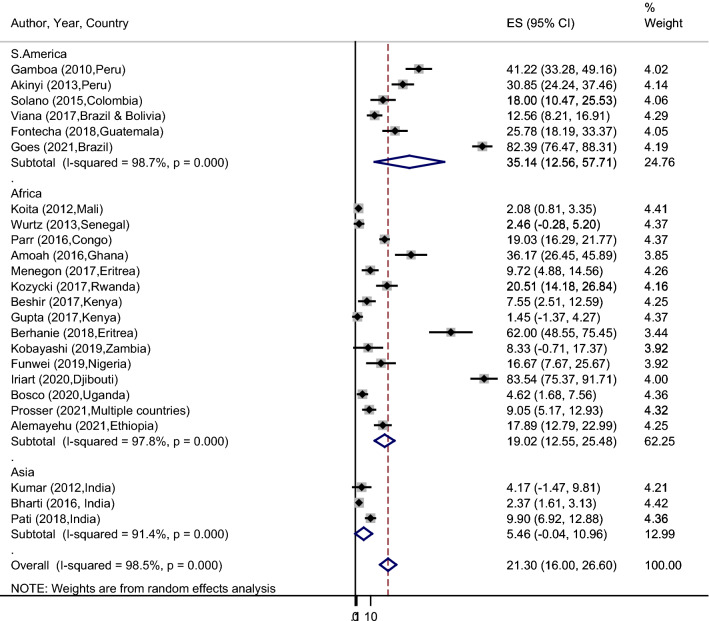
Forest plot representing subgroup analysis of pooled *pfhrp2* gene deletion by continent

Subgroup analysis based on continent showed that the pooled prevalence of PfHRP2 gene deletion in South America, Africa, and Asia were 35.14% (95% CI 12.56–57.71%), 19.02% (95% CI 12.56–25.48%), and 5.46% (95% CI 0.04–10.96%), respectively (Fig. 6). Similar pattern was also observed for PfHRP3 gene deletion analysis and therefore 62.00%, 29.10%, and 3.89% were seen in South America, Africa, and Asia, respectively.

This meta-analysis study also showed that the pooled estimated values of double PfHRP2/3 gene deletion was 18.65%. The summary estimates based on the different types of PfHRP genes deletion is presented in Table 2.

**Table 2:** Summary estimates for pooled pfhrp gene deletions based on gene types

Type of *pfhrp* deletions	Number of studies included	Pooled estimate (95% Cl)	*I*^2^ (%)
*Pfhrp2*	24	21.29 (15.99, 26.60)	98.5
*Pfhrp3*	17	34.49 (20.38, 48.61)	99.7
Double	11	18.65(12.40, 24.89)	97.9

### Proportion of PfHRP2 gene deletions among PfHRP*2*-based RDT false negatives

A total of 10 studies were included to determine the estimated pooled proportion of PfHRP2 gene deletions among PfHRP2*-*based RDT false negatives. Although additional four studies (1 from Eritrea, 1 from Kenya, 1 from Swaziland and 1 from India) have determined the proportion of PfHRP2 gene deletion, they were not included in the meta-analysis following sensitivity analysis [17, 22, 26, 28]. The proportion of PfHRP2 deletions among the PfHRP2-based RDT false negative cases by crude analysis ranged from 1.4 to 92.41%, while the estimated proportions across the studies using meta-analysis were within the range of 35.20–45.61%. The estimated pooled proportion of this gene deletion was 41.10% (95% CI 21.13–61.07%) (Table 3).

**Table 3:** Estimated proportions of pfhrp2 gene deletions among false negative cases using PfHRP2-based RDTs

Authors	Year of Publication	Country	# confirmed PF, but Negative *pfhrp2*-based RDT	Number and proportion of *pfhrp2*-using crude analysis	Estimate (95% conf. interval) using meta-analysis
Bharti et al. [50]	2016	India	50	36 (72.00)	37.73 (17.01–58.44)
Parr et al. [36]	2016	Congo	783	149 (19.00)	43.62 (15.57–71.66)
Kozycki et al. [41]	2017	Rwanda	140	48 (34.28)	41.87 (20.02–63.72)
Amoah et al. [39]	2016	Ghana	38	6 (15.80)	43.88 (22.45–65.31)
Gupta et al. [42]	2016	Mozambique	69	1 (1.40)	45.61 (22.73–68.48)
Kobayashi et al. [43]	2019	Zambia	36	3 (8.30)	44.75 (23.14–66.37)
Pati et al. [49]	2018	India	58	38 (65.50)	38.43 (17.55–59.31)
Funwei et al. [44]	2019	Nigeria	31	7 (22.60)	43.09 (21.82–64.36)
Iriart et al. [30]	2020	Djibouti	66	61 (92.42)	35.20 (19.24–51.16)
Alemayehu et al. [48]	2021	Ethiopia	86	69 (80.23)	36.71 (17.04–56.38)
Combined	–	All the above countries	1357	418 (30.80)	41.10 (21.13–61.07)

### Approaches of PfHRP2 gene deletion analysis by the individual studies

As it is presented in Table 4 below, only 8.3% (2/24) of the individual studies have followed the recently published WHO PfHRP2 gene deletion analysis protocol. Accordingly, the subgroup analysis was done based on whether they were followed the guideline or not. Thus, the pooled estimate of PfHRP2 gene deletion among studies which did not follow the protocol was 21.3% (95% CI 15.91–29.17%), whereas among those who have followed the guideline was 10.5% (95% CI − 6.10 to 27.12). All studies have used nested PCR as a methods of PfHRP2 gene deletion detection. PCR amplification of DNA fragments encompassing exon1, intron, exon2, and fragment encompassing exon2 of PfHRP2 and PfHRP3 genes were performed using specific primers for confirmation of deletion of these genes [29].

**Table 4:** Approaches of pfhrp2 gene deletion analysis against the WHO guideline for its investigation

Author	Study design	Participants	Sample size	Type of samples	Pf Confirmation	Methods of pfhrp2 gene deletion detection
WHO guideline	Cross sectional	Symptomatic	370	RDT − and Microscopy + or RDT − and Pf-pLDH RDT +	PCR	PCR
Koita^a^ et al. [16]	Retrospective	Symptomatic	480	HRP2 RDT − and Microscopy +	PCR	PCR
Wurtz^b^ et al. [38]	Cross sectional	Symptomatic	122	HRP2 RDT − and Microscopy +	PCR	PCR
Parr^a^ et al. [36]	Cross sectional	Asymptomatic	783	RDT −/PCR +	PCR	PCR
Amoah^b^ et al. [39]	Cross sectional	Healthy children	94	RDT −/PCR +	PCR	PCR
Menegon^b^ et al. [40]	Cross sectional	Symptomatic	144	Microscopy + /PCR +	PCR	PCR
Kozycki^b^ et al. [41]	Cross sectional	Symptomatic	156	RDT − and Microscopy +	PCR	PCR
Beshir^b^ et al. [19]	Cross sectional	Asymptomatic	106	PCR +	PCR	PCR
Gupta^b^ et al. [42]	Cross sectional	All population	69	RDT − and Microscopy +	PCR	PCR
Berhanie^b^ et al. [22]	Cross sectional	Symptomatic	50	RDT − and Microscopy +	PCR	PCR
Kobayashi^b^ et al. [43]	Retrospective	Asymptomatic	36	RDT − and Microscopy +	PCR	PCR
Funwei^b^ et al. [44]	Not specified	Symptomatic	66	RDT − and Microscopy +	PCR	PCR
Iriart^b^ et al. [30]	Cross sectional	Symptomatic	79	RDT −/PCR +	PCR	PCR
Bosco^b^ et al. [46]	Cross sectional	Symptomatic	195	RDT −/PCR +	PCR	PCR
Prosser^b^ et al. [47]	Retrospective	Symptomatic	210	PCR +	PCR	PCR
Alemayehu^b^ et al. [48]	Cross sectional	Symptomatic	218	PCR +	PCR	PCR
Pati^a^ et al. [49]	Cross sectional	Symptomatic	384	RDT −/PCR +	PCR	PCR
Bharti^b^ et al. [50]	Cross sectional	Symptomatic	1521	PCR +	PCR	PCR
Kumar^b^ et al. [17]	Cross sectional	Symptomatic	48	Microscopy +/PCR +	PCR	PCR
Viana et al. [51]	Cross sectional	Symptomatic	223	Microscopy +/PCR +	PCR	PCR
Gamboa^b^ et al. [13]	Retrospective	Symptomatic	148	Microscopy +	PCR	PCR
Goes^b^ et al. [52]	Retrospective	Symptomatic	159	Microscopy +/PCR +	PCR	PCR
Akinyi^b^ et al. [53]	Retrospective	Symptomatic	188	Microscopy +/PCR +	PCR	PCR
Solano^b^ et al. [12]	Retrospective	Symptomatic	100	Not clear	PCR	PCR
Fontecha^b^ et al. [54]	Cross sectional	Symptomatic	128	Microscopy +/PCR +	PCR	PCR

^a^studies which have followed the WHO pfhrp2 gene deletion analysis protocol

^b^studies which did not follow the WHO pfhrp2 gene deletion analysis protocol

## Discussion

Evidence on the pooled estimates of PfHRP2/3 gene deletions is limited in the global context. Based on the individual articles that are included under this review, the level of PfHRP2 gene deletion across malaria endemic countries ranged from the highest 100% (locality in Ethiopia) to the lowest 0% (locality in Swaziland) [26, 27]. However, these two outlier individual studies were not included in the meta-analysis. This study showed that, the pooled prevalence of PfHRP2 gene deletion across malaria endemic countries ranged from the highest 83.54% (locality in Djibouti) to the lowest 1.45% (2 localities Kenya) [19, 30]. Moreover, the overall prevalence of malaria parasite with PfHRP2 gene deletion is relatively very high (21%). Even though most of the individual studies have not adhered to the WHO PfHRP2 gene deletion investigation protocol (Table 4), the pooled estimate of the PfHRP2 gene deletion in both groups or studies which did/did not follow the guideline surpasses the WHO threshold level so as to switch to an alternative malaria diagnostic tools (10.5% vs 21.3%) [23]. These findings are higher than those of the findings from previous studies. For example, a systematic review and meta-analysis done by Sepulvada et al. suggested a 17% pooled prevalence of PfHRP2 gene deletion among *P. falciparum* confirmed isolates [31]. Furthermore, a study conducted in Africa and India showed 8% and 5% of the gene deletion, respectively [32]. The observed differences in the levels of PfHRP2 gene deletion in the systematic review and meta-analysis studies could be due to selection pressure caused by exclusive use of PfHRP2 based RDTs over time as suggested in previous studies [22, 33]. Moreover, it might be attributable to the differences in study design, period of sampling and study populations.

In this study, there was a significant heterogeneity among studies and hence a subgroup analysis was carried out. Accordingly, the continent-wise subgroup analysis showed that the pooled prevalence of PfHRP2 gene deletions are higher in South America than from other continents, followed by Africa and Asia. The observed differences might be associated with the study period and the level of parasite transmission intensity. The other subgroup analysis of this review was based on the study design, and the pooled PfHRP2 gene deletion was higher among retrospective studies (27.4%) than the cross-sectional studies (20.4%). This might be related to the gradual deterioration of amino acids coded for PfHRP2 genes since they have used stored samples and this may ultimately result in false negative PfHRP2 genes from such historical samples. However, the prevalence of this gene deletion in all subgroups is above the WHO minimum threshold level for switching to another alternative malaria diagnostic tools.

This study has also determined the proportion of PfHRP2 gene deletions among false negative results using PfHRP2 based RDTs. Although the individual studies showed this gene deletion within the range of 1.4–92.41%, the estimated pooled proportion across the studies was 41.10% (95% CI 21.13–61.71%). This high prevalence of PfHRP2 deletion threatens the use of PfHRP2-based RDT for malaria diagnosis. As a consequence of false PfHRP2-based RDTs, malaria patients will be exposed to drugs used for treating co-endemic viral and bacterial infectious diseases which have symptomatology similar to that of malaria [34].

A protein encoded by PfHRP3 gene has the capacity to cross react with monoclonal antibodies of RDTs targeting PfHRP2 and hence reduce false negative results. However, high level of PfHRP3 gene deletion was also found in this review. Its deletion ranged from 1.78 to 96.23% across the individual studies with an overall prevalence of 34.5% (Fig. 6). This finding is higher than the previous studies conducted in Africa and India [32].

This systematic review and meta-analysis showed that there is an increasing pattern of PfHRP2 gene deletion over time and space. For example, a review which had included studies conducted from 2010 to 2017 showed that the pooled prevalence was 8% [32]. On the other hand, the pooled estimated prevalence of PfHRP2 gene deletion in the current study (studies conducted from 2010 to 2021) is 21.3%. This significant increment might be related with the unrestricted use of PfHRP2-based RDTs that misses PfHRP2 negative strains leading to their continued circulation [22, 33]. This selection pressure and spontaneous occurrence of gene-deleted parasites has been predicted by recent mathematical modelling, that showed exclusive use of PfHRP2-based RDTs exerts strong selection pressure for PfHRP2/3-negative parasite populations leading to their increase in frequency [35]. This finding may enforce utilization of RDTs targeting several plasmodia antigens simultaneously instead of RDTs targeting PfHRP2.

As a limitation of this study, studies which did and did not comply the WHO guideline for PfHRP2 gene deletion analysis were included in the analysis and only two studies have followed the standard procedure [16, 36]. On the other hand, the rest studies failed to fulfil at least one of the stringent criteria of the protocol. For example, some studies used asymptomatic study population, and some others used insufficient sample size (Table 4). All these issues have resulted in a high heterogeneity in the meta-analysis, and this is inescapable in meta-analyses of prevalence and observational studies [37]. Therefore, all these may affect the quality of this systematic review and meta-analysis and should be interpreted with caution.

## Conclusion

This systematic review and meta-analysis showed that there is a high prevalence of PfHRP2 and PfHRP3 gene deletions, indicating the impending challenges in the use of this crucial tool in malaria control programs. This study underlined that PfHRP2-based diagnosis of falciparum malaria remain under question. Thus, further studies with standardized approaches in order to have a clearer picture of the extent of mutants with deletions in PfHRP2/3 genes and follow their patterns over time and space is recommended.

## Data Availability

We confirm that all the data for this manuscript are available and can be shared upon request. Requests can be directed to the first author (AJZ).

## Abbreviations

HRP: Histidine-rich protein-2
RDTs: Rapid diagnostic tests
PRISMA: Preferred reporting items for systematic reviews and meta-analyses guidelines
WHO: World Health Organization
